# Magnesium depletion score is a risk factor for all-cause, cancer and cardiovascular disease mortality in cancer survivors: evidence from two prospective cohort studies

**DOI:** 10.3389/fnut.2025.1674062

**Published:** 2025-10-31

**Authors:** Yunmiao Ma, Minjie Li, Xing Liu

**Affiliations:** ^1^Department of Oncology, Shaanxi Provincial People's Hospital, Xi’an, China; ^2^Department of Nursing, Shaanxi Provincial People's Hospital, Xi’an, China; ^3^Department of Urology, Shaanxi Provincial People's Hospital, Xi’an, China

**Keywords:** cancer survivors, magnesium deficiency, magnesium depletion score, cause-specific mortality, prognosis

## Abstract

**Background:**

Cancer survivors face significant mortality risks, including from cardiovascular disease (CVD) and cancer. Magnesium depletion score (MDS) is a prognostic biomarker, but its prognostic value in cancer survivors is unknown.

**Methods:**

This prospective study utilizes data from two distinct cohorts: the National Health and Nutrition Examination Survey (NHANES) and the Shaanxi Provincial People’s Hospital. We analyzed 3,528 cancer survivors from NHANES (1999–2018) and 473 patients from the hospital cohort. Associations between MDS (0, 1, 2, ≥3) and all-cause, cancer-specific, and CVD mortality were assessed using weighted multivariate Cox regression, Kaplan–Meier analysis and Fine-Gray competing risk model. Predictive accuracy was evaluated using time-dependent ROC curves.

**Results:**

During follow-up (median 165 months for NHANES), higher MDS (≥3) was significantly associated with increased mortality risk after full adjustment: all-cause (HR = 2.48, 95% CI: 1.86–3.29), cancer (HR = 2.17, 95% CI: 1.44–3.26), and CVD (HR = 3.89, 95% CI: 1.91–7.91) compared to MDS = 0 (all *p* for trend<0.001). Kaplan–Meier curves and competing risk model confirmed worse survival with higher MDS. Time-dependent ROC demonstrated good predictive accuracy for all mortality risk. Results remained robust in sensitivity analyses and the single-center cohort.

**Conclusion:**

Higher MDS is a strong, independent predictor of increased all-cause, cancer-specific, and CVD mortality risk among cancer survivors. MDS provides valuable prognostic information and could aid in risk stratification for personalized survivorship care.

## 1 Background

Cancer has become a significant global burden, with an estimated 20 million new cases and 9.7 million deaths worldwide in 2022 (1). The incidence is projected to increase to over 27 million cases annually by 2040, with the greatest proportional increase in low- and middle-income countries (2). Global cancer statistics indicate that approximately one in five individuals will develop cancer in their lifetime, with about one in nine men and one in 12 women dying from the disease (1). In the United States, the lifetime risk is even higher, with about 40% of men and women expected to be diagnosed with cancer (3). The number of cancer survivors in the United States has grown substantially, with projections indicating further increases (4). Cancer survivors face numerous challenges, including physical, psychosocial, cognitive issues, and cardiovascular diseases (CVD), as well as concerns about recurrence and new malignancies (5, 6). Therefore, personalized survivorship management plans and studies into biomarkers are essential for addressing the diverse needs of this population.

Prognostic indicators play a crucial role in predicting outcomes for cancer survivors. Numerous studies have identified serum magnesium as a significant predictive biomarker for cancer prognosis. Xu et al. (7) demonstrated that higher magnesium levels in non-small cell lung cancer patients undergoing epidermal growth factor receptor tyrosine kinase inhibitor (EGFR-TKI) therapy correlated with improved progression-free survival and overall survival. Similarly, Reis et al. (8) found that altered serum magnesium levels were robust predictors of mortality in critically ill cancer patients. Collectively, these findings underscore the importance of serum magnesium as a prognostic factor for cancer survivors.

Magnesium deficiency assessment is challenging, as serum magnesium concentration, the most commonly used method, does not reliably reflect total body magnesium levels or tissue concentrations (9). A normal serum magnesium level can mask chronic latent magnesium deficiency, as bone magnesium supplements blood levels (10). The magnesium depletion score (MDS) is a newly developed tool designed to evaluate magnesium deficiency by combining several clinical factors, including diuretic use, proton pump inhibitor use, estimated glomerular filtration rate (eGFR), and alcohol abuse (11). However, the role of MDS in prognostic prediction for cancer survivors is not yet established.

Leveraging National Health and Nutrition Examination Survey (NHANES) data, our study examined associations between MDS and all-cause, cancer, and CVD mortality in cancer survivors. These findings highlight the clinical relevance of monitoring MDS in this population.

## 2 Methods and materials

### 2.1 Study population

The present study utilized data from two complementary sources. The NHANES is a comprehensive, ongoing health survey of the United States population conducted by the National Center for Health Statistics (NCHS) (12). This nationally representative survey employs a multistage probability sampling framework, incorporating structured household interviews, standardized physical examinations, and biological specimen banking (13). All NHANES protocols received institutional review board authorization, with participants providing documented consent for genetic analyses and biobanking. Our analytical cohort comprised adult cancer survivors from NHANES 1999–2018. Individuals lacking complete data on MDS or key covariates were excluded. Additionally, the study included 473 cancer patients diagnosed and treated at Shaanxi Provincial People’s Hospital between May 2018 and January 2019. Baseline characteristics, laboratory data and cause-specific mortality were collected retrospectively from institutional electronic medical records (EMR). Ethical oversight was maintained through approvals from Shaanxi Provincial People’s Hospital Ethics Committee (No.: 2022K003) and adherence to Helsinki Declaration principles.

### 2.2 Mortality follow-up

This investigation defined primary endpoints as all-cause mortality, cancer-specific mortality, and CVD mortality. The NCHS has created linked mortality files (up to December 31, 2019) for NHANES by connecting survey data to the National Death Index (NDI) (14). The mortality follow-up data of the single center participants were collected from Shaanxi Provincial People’s Hospital EMR.

### 2.3 Assessment of MDS

The MDS is a novel metric for quantifying magnesium deficiency risk based on four clinical parameters (15): (1) diuretic use (score 1); (2) proton pump inhibitor intake (score 1); (3) alcohol consumption (score 1 if more than 1 drink/day for females or 2 drinks/day for males); and (4) eGFR (score 1 if values are 60–90 mL/min/1.73 m^2^ or score 2 if <60 mL/min/1.73 m^2^). Due to insufficient subjects with higher scores, previous studies consistently categorized MDS into four groups (0, 1, 2, ≥3) when utilizing it as a categorical variable (16, 17).

### 2.4 Covariates collection

The study collected comprehensive baseline characteristics encompassing sociodemographic factors (age, sex, self-reported race, educational attainment, poverty-income ratio [PIR], marital status), body mass index (BMI), and behavioral indicators (smoking/drinking status). Diagnosis of comorbid conditions—including CVD, diabetes mellitus (DM), chronic kidney disease (CKD), metabolic syndrome (MetS), hypertension, and dyslipidemia—relied on standardized laboratory assessments. Detailed definitions of above covariates are documented in Supplementary Table 1.

### 2.5 Statistical analyses

For the single center data, continuous variables were reported as mean and standard deviation (SD), while categorical variables were expressed as numbers and percentages. For NHANES data, all analyses were conducted in accordance with the NHANES statistical analysis protocol and included the application of NHANES sampling weights. Significant differences were identified using t-tests for continuous variables and chi-squared tests for categorical variables. Continuous variables were presented as mean ± standard error (SE), while categorical variables were showed as counts and weighted percentages. The weighted multivariate cox regression analysis and Kaplan–Meier curve were performed to evaluate the effect of different MDS groups on mortality outcomes. The crude model was not adjusted for variable. Model 1 included adjustments for age, sex, race, and education level, while Model 2 further adjusted for marital status, PIR, BMI, smoking, drinking, hypertension, hyperlipidemia, DM, CVD, CKD, and MetS. Then, cumulative incidence function curves of competition risk model were constructed using Fine-Gray’s test to eliminate the influence of competitive risk event (18). The time-dependent receiver operating characteristic (ROC) curve analysis was performed to assess the accuracy of MDS in survival outcome prediction using the timeROC package (19). All statistical analyses were conducted using R software, with a *p* value of less than 0.05 considered to be statistically significant.

## 3 Results

### 3.1 Baseline characteristics of included individuals

**Figure 1:**
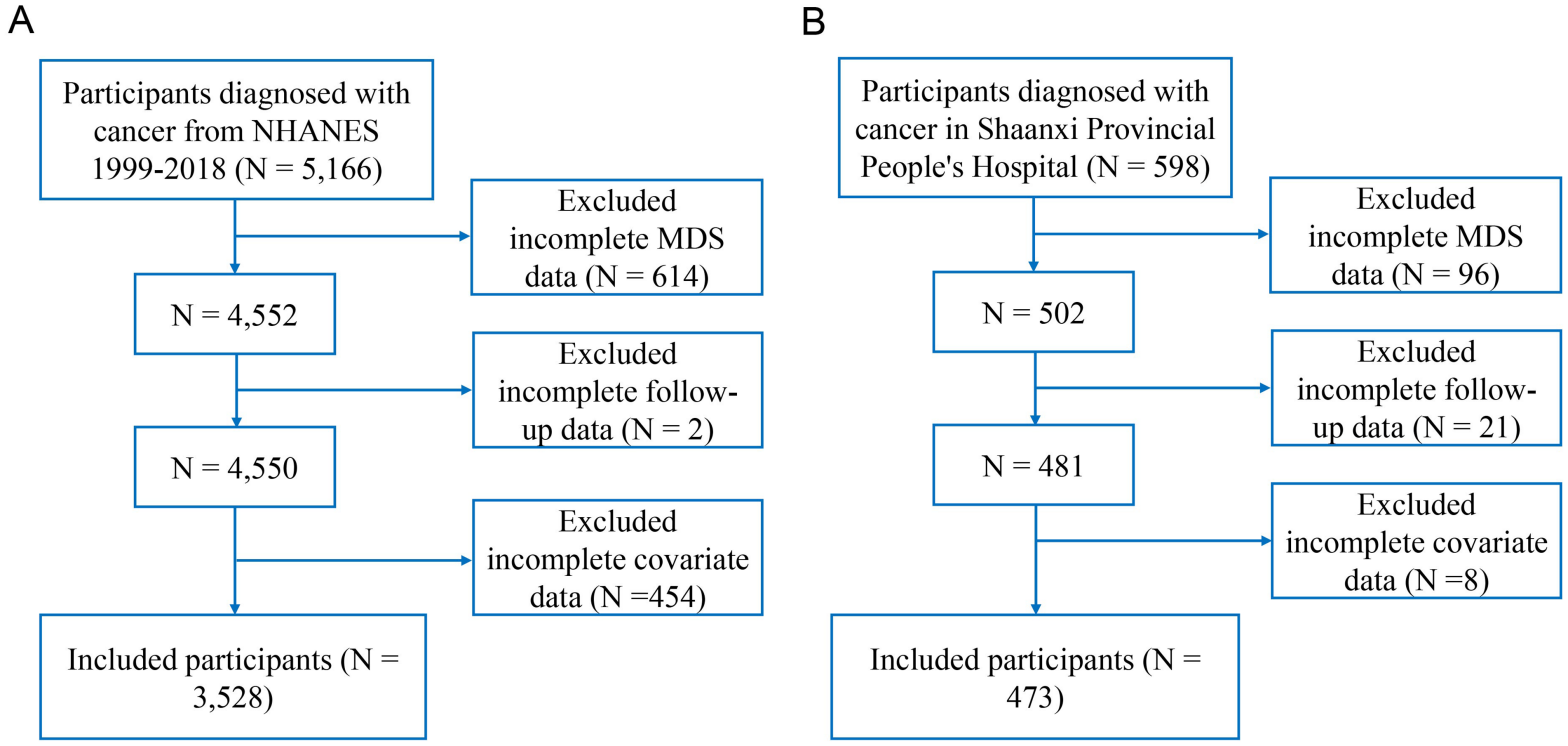
Flow chart of study cohort selection. **(A)** NHANES, **(B)** the single center. MDS, magnesium depletion score; NHANES, National Health and Nutrition Examination Survey.

The analytical cohorts comprised 3,528 eligible NHANES participants (1,265 all-cause deaths during follow-up) and 473 hospital patients (244 deaths) from Shaanxi Provincial People’s Hospital. The flowchart of participant selection was shown in Figure 1. The baseline characteristics of the participants from NHANES and single center are presented in Tables 1, 2. The average age of the patients from the single center was 71.10 years, whereas the average age of the NHANES participants was 62.15 years. Notably, NHANES individuals with higher MDS demonstrated higher age and BMI, lower education level and eGFR, increased comorbidities (CVD, DM, CKD, hypertension, hyperlipidemia, and MetS), as well as elevated mortality. Similarly, the single center individuals with higher MDS showed higher age, lower eGFR, increased comorbidities (CVD, CKD, hypertension, hyperlipidemia, and MetS), as well as elevated mortality.

**Table 1:** Weighted clinical features of NHANES participants based on different MDS group.

Variables	Total	MDS group				*P*-value
		0	1	2	≥3	
Age, year	62.15 ± 0.34	49.17 ± 0.74	61.53 ± 0.44	67.56 ± 0.49	72.45 ± 0.53	<0.0001
Age group						<0.0001
<50	505(19.37)	274(47.77)	175(18.55)	43(7.34)	13(2.81)	
≥50	3,023(80.63)	366(52.23)	1,100(81.45)	951(92.66)	606(97.19)	
Sex						<0.0001
Female	1832(57.19)	419(67.81)	635(53.80)	466(54.10)	312(56.40)	
Male	1,696(42.81)	221(32.19)	640(46.20)	528(45.90)	307(43.60)	
BMI						<0.001
<25	1,019(29.91)	189(32.80)	423(33.68)	252(25.28)	155(24.26)	
25–30	1,244(34.19)	198(29.49)	435(33.36)	392(39.09)	219(34.32)	
≥30	1,265(35.91)	253(37.71)	417(32.96)	350(35.63)	245(41.42)	
Race						<0.0001
White	2,555(88.30)	364(81.70)	932(89.38)	756(90.07)	503(91.68)	
Mexican American	229(1.90)	83(3.88)	82(1.79)	49(1.21)	15(0.59)	
Black	453(4.78)	104(6.20)	156(4.23)	119(4.30)	74(5.06)	
Other	291(5.01)	89(8.21)	105(4.59)	70(4.42)	27(2.68)	
Education level						0.01
College or above	1954(64.18)	361(68.43)	722(64.54)	551(63.63)	320(58.25)	
High school or equivalent	809(22.15)	142(19.87)	302(23.66)	218(21.33)	147(22.88)	
Less than High school or equivalent	765(13.68)	137(11.69)	251(11.80)	225(15.04)	152(18.87)	
PIR						<0.0001
0–1.3	810(15.22)	205(20.56)	262(13.69)	208(12.93)	135(15.67)	
1.3–3.5	1,446(36.23)	217(30.13)	511(33.50)	431(39.52)	287(46.01)	
>3.5	1,272(48.55)	218(49.30)	502(52.81)	355(47.55)	197(38.32)	
Hypertension						<0.0001
No	1,260(42.34)	378(67.70)	545(47.86)	263(30.86)	74(12.83)	
Yes	2,268(57.66)	262(32.30)	730(52.14)	731(69.14)	545(87.17)	
Hyperlipidemia						<0.0001
No	678(18.98)	173(28.07)	254(19.35)	166(14.98)	85(12.37)	
Yes	2,850(81.02)	467(71.93)	1,021(80.65)	828(85.02)	534(87.63)	
CVD						<0.0001
No	2,667(80.84)	565(91.06)	1,041(85.40)	708(77.02)	353(61.57)	
Yes	861(19.16)	75(8.94)	234(14.60)	286(22.98)	266(38.43)	
DM						<0.0001
No	2,296(70.03)	467(78.54)	882(73.35)	601(64.68)	346(58.96)	
IFG	199(5.81)	30(4.83)	69(5.17)	68(7.69)	32(5.56)	
IGT	141(3.44)	22(2.14)	53(3.93)	40(3.49)	26(3.89)	
DM	892(20.72)	121(14.49)	271(17.54)	285(24.14)	215(31.59)	
CKD						<0.0001
No	2,397(75.68)	561(91.39)	1,074(89.00)	592(67.35)	170(34.18)	
Yes	1,131(24.32)	79(8.61)	201(11.00)	402(32.65)	449(65.82)	
eGFR	82.15 ± 0.43	103.88 ± 0.42	86.14 ± 0.50	73.12 ± 0.68	58.36 ± 1.00	<0.0001
MetS						<0.0001
No	2058(61.50)	473(77.31)	797(63.95)	521(55.24)	267(44.11)	
Yes	1,470(38.50)	167(22.69)	478(36.05)	473(44.76)	352(55.89)	
Drinking						<0.0001
No	1,316(29.92)	215(25.36)	454(27.38)	388(32.64)	259(38.07)	
Yes	2,212(70.08)	425(74.64)	821(72.62)	606(67.36)	360(61.93)	
Smoke						<0.0001
Never	1,535(44.73)	293(45.57)	573(45.43)	403(41.73)	266(46.98)	
Former	1,459(39.29)	176(28.27)	494(36.69)	482(47.51)	307(47.01)	
Now	534(15.98)	171(26.15)	208(17.88)	109(10.76)	46(6.01)	
Outcome						<0.0001
Alive	2,263(74.13)	531(89.03)	869(77.48)	588(68.67)	275(54.26)	
Dead	1,265(25.87)	109(10.97)	406(22.52)	406(31.33)	344(45.74)	

Mean (SE) for continuous variables; number (%) for categorical variables. Differences between groups were analyzed by nonparametric t-test or chi-squared test.

BMI, body mass index; CKD, chronic kidney disease; CVD, cardiovascular disease; DM, diabetes mellitus; IFG, impaired fasting glucose; IGT, impaired glucose tolerance; MetS, metabolic syndrome; MDS, magnesium depletion score; PIR, Poverty-income ratio.

**Table 2:** Clinical features of the single center participants based on different MDS group.

Variables	Total	MDS group				*P*-value
		0	1	2	≥3	
Age, year	71.10 ± 12.66	53.1 ± 16.1	69.9 ± 11.3	75.0 ± 9.2	76.3 ± 7.0	<0.01
Age group						<0.01
<50	37(7.82)	25(43.86)	7(4.70)	4(2.84)	1(0.79)	
≥50	436(92.18)	32(56.14)	142(95.30)	137(97.16)	125(99.21)	
Sex						0.11
Female	202(42.71)	33(57.89)	61(40.94)	57(40.43)	51(40.48)	
Male	271(57.29)	24(42.11)	88(59.06)	84(59.57)	75(59.52)	
BMI						0.32
<25	151(31.92)	15(26.32)	49(32.89)	45(31.91)	42(33.33)	
25–30	167(35.31)	25(43.86)	55(36.91)	40(28.37)	47(37.30)	
≥30	155(32.77)	17(29.82)	45(30.20)	56(39.72)	37(29.37)	
Hypertension						<0.01
No	154(32.56)	34(59.65)	58(38.93)	42(29.79)	20(15.87)	
Yes	319(67.44)	23(40.35)	91(61.07)	99(70.21)	106(84.13)	
Hyperlipidemia						0.02
No	96(20.30)	20(35.09)	28(18.79)	29(20.57)	19(15.08)	
Yes	377(79.70)	37(64.91)	121(81.21)	112(79.43)	107(84.92)	
CVD						<0.01
No	296(62.58)	52(91.23)	108(72.48)	80(56.74)	56(44.44)	
Yes	177(37.42)	5(8.77)	41(27.52)	61(43.26)	70(55.56)	
DM						0.08
No	279(58.99)	37(64.91)	97(65.10)	81(57.45)	64(50.79)	
DM	194(41.01)	20(35.09)	52(34.90)	60(42.55)	62(49.21)	
CKD						<0.01
No	241(50.95)	44(77.19)	112(75.17)	63(44.68)	22(17.46)	
Yes	232(49.05)	13(22.81)	37(24.83)	78(55.32)	104(82.54)	
eGFR	72.24 ± 22.78	104.70 ± 9.30	83.88 ± 11.76	67.76 ± 18.74	51.44 ± 16.74	<0.01
MetS						<0.01
No	263(55.60)	43(75.44)	89(59.73)	74(52.48)	57(45.24)	
Yes	210(44.40)	14(24.56)	60(40.27)	67(47.52)	69(54.76)	
Drinking						0.17
No	171(36.15)	15(26.32)	51(34.23)	51(36.17)	54(42.86)	
Yes	302(63.85)	42(73.68)	98(65.77)	90(63.83)	72(57.14)	
Smoke						0.02
Never	219(46.30)	20(35.09)	66(44.30)	66(46.81)	67(53.17)	
Former	184(38.90)	22(38.60)	55(36.91)	58(41.13)	49(38.89)	
Now	70(14.80)	15(26.32)	28(18.79)	17(12.06)	10(7.94)	
Outcome						<0.01
Alive	229(48.41)	40(70.18)	80(53.69)	59(41.84)	50(39.68)	
Dead	244(51.59)	17(29.82)	69(46.31)	82(58.16)	76(60.32)	

Mean (SD) for continuous variables; number (%) for categorical variables. Differences between groups were analyzed by nonparametric *t*-test or chi-squared test.

BMI, body mass index; CKD, chronic kidney disease; CVD, cardiovascular disease; DM, diabetes mellitus; MetS, metabolic syndrome; MDS, magnesium depletion score.

### 3.2 Association between MDS and all-cause, cancer, and CVD mortality

Over a 165-month median follow-up in NHANES, we documented 394 cancer-specific and 276 CVD deaths. The weighted multivariable cox regression models were performed to explore the association between different MDS groups and all-cause, cancer, and CVD mortality (Table 3). HR values were visualized by forest plot (Supplementary Figure 1). After full covariates adjustment, individuals with MDS ≥ 3 exhibited a significant higher risk of all-cause (HR, 95% CI: 2.48, 1.86–3.29), cancer (HR, 95% CI: 2.17, 1.44–3.26) and CVD mortality (HR, 95% CI: 3.89, 1.91–7.91) compared to those with MDS = 0. Furthermore, we carried out an additional trend analysis to examine the relationship between MDS and the mortality risk. Trend analyses confirmed monotonically increasing mortality risks with ascending MDS scores (all *p* for trend < 0.001).

**Table 3:** Hazard ratio of all-cause, cancer, and CVD mortality among participants with different MDS groups.

Cause of death	MDS groups, HR (95% CI)				*p*	*p* for trend
	0	1	2	≥3		
All-cause						
Crude model	Ref	2.05(1.58,2.65)	3.07(2.37,3.98)	5.77(4.43,7.51)	<0.0001	<0.0001
Model 1	Ref	1.32(1.00,1.76)	1.71(1.32,2.21)	3.06(2.36,3.98)	<0.0001	<0.0001
Model 2	Ref	1.30(0.99,1.71)	1.54(1.20,1.97)	2.48(1.86,3.29)	<0.0001	<0.0001
Cancer						
Crude model	Ref	1.35(0.88,2.05)	2.12(1.41,3.19)	3.92(2.59,5.92)	<0.0001	<0.0001
Model 1	Ref	0.88(0.57,1.34)	1.19(0.79,1.77)	2.19(1.42,3.38)	<0.001	<0.0001
Model 2	Ref	0.95(0.64,1.40)	1.20(0.83,1.73)	2.17(1.44,3.26)	<0.001	<0.001
CVD						
Crude model	Ref	3.25(1.71, 6.20)	5.56(2.79,11.08)	13.73(6.90,27.33)	<0.0001	<0.0001
Model 1	Ref	1.84(0.95, 3.57)	2.60(1.28, 5.29)	6.04(3.02,12.09)	<0.0001	<0.0001
Model 2	Ref	1.74(0.88, 3.41)	1.98(0.97, 4.04)	3.89(1.91, 7.91)	<0.001	<0.0001

Crude model: unadjusted.

Model 1: adjusted for age, sex, race, education level.

Model 2: further adjusted for marital status, PIR, BMI, smoking, drinking, hypertension, hyperlipidemia, DM, CVD, CKD, and MetS.

BMI, body mass index; CI, confidence interval; CKD, chronic kidney disease; CVD, cardiovascular disease; DM, diabetes mellitus; MetS, metabolic syndrome; MDS, magnesium depletion score; HR, hazard ratio; PIR, Poverty-income ratio; Ref, reference.

**Figure 2:**
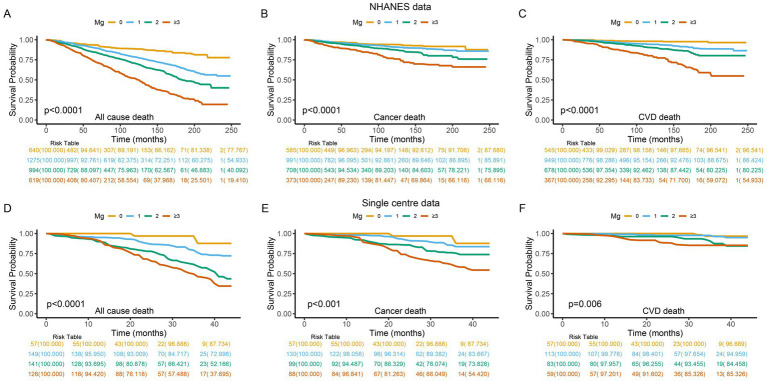
The overall survival Kaplan–Meier curves for the mortality risk of all-cause **(A,D)**, cancer **(B,E)**, and CVD **(C,F)** stratified by MDS group. The survival curve showed that patients with MDS ≥ 3 had a worse survival rate across all-cause, cancer, and CVD mortality. CVD, cardiovascular disease; MDS, magnesium depletion score.

**Figure 3:**
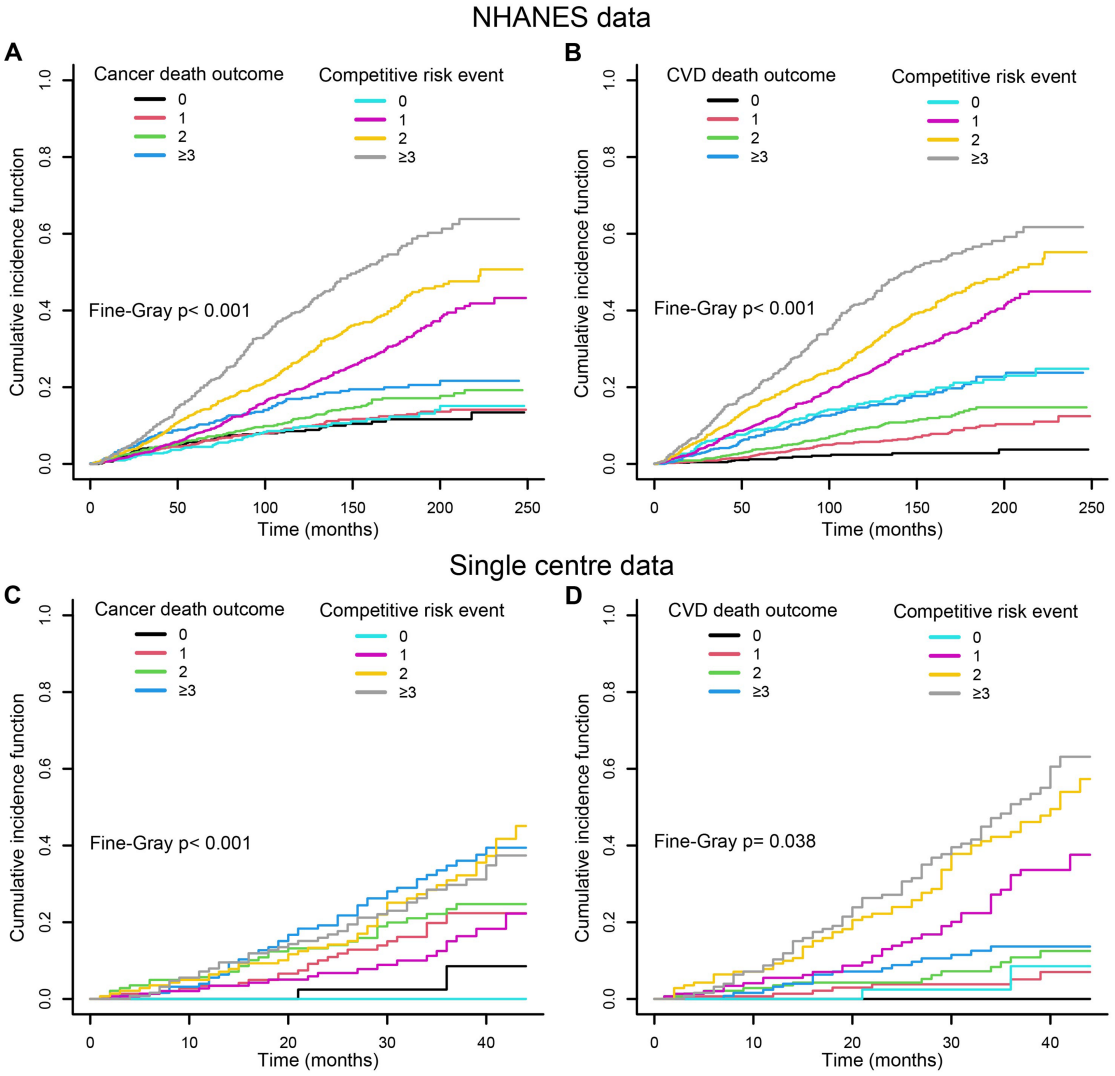
Association between MDS and cancer **(A,C)** or CVD **(B,D)** mortality with non-cancer or non-CVD mortality as competing risk, respectively. The results indicated that when competitive risk events were controlled, the higher MDS were significantly positive associated with the risk of cancer and CVD mortality. CVD, cardiovascular disease; MDS, magnesium depletion score.

Kaplan–Meier analysis demonstrated significant differences in survival outcomes when stratifying participants by MDS categories. The survival curve showed that patients with MDS ≥ 3 had a worse survival rate across all-cause, cancer, and CVD mortality (Figure 2). Subsequently, we performed the competitive risk model to further explore the contribution of MDS to mortality risk. A competitive risk model was constructed using the Fine-Gray test with cancer or CVD-related deaths as the outcome, and non–outcome related deaths as the competitive risk event. The results indicated that when competitive risk events were controlled, the higher MDS were significantly positive associated with the risk of cancer and CVD mortality (Figure 3).

### 3.3 ROC analysis of MDS for predicting mortality risk in cancer patients

**Figure 4:**
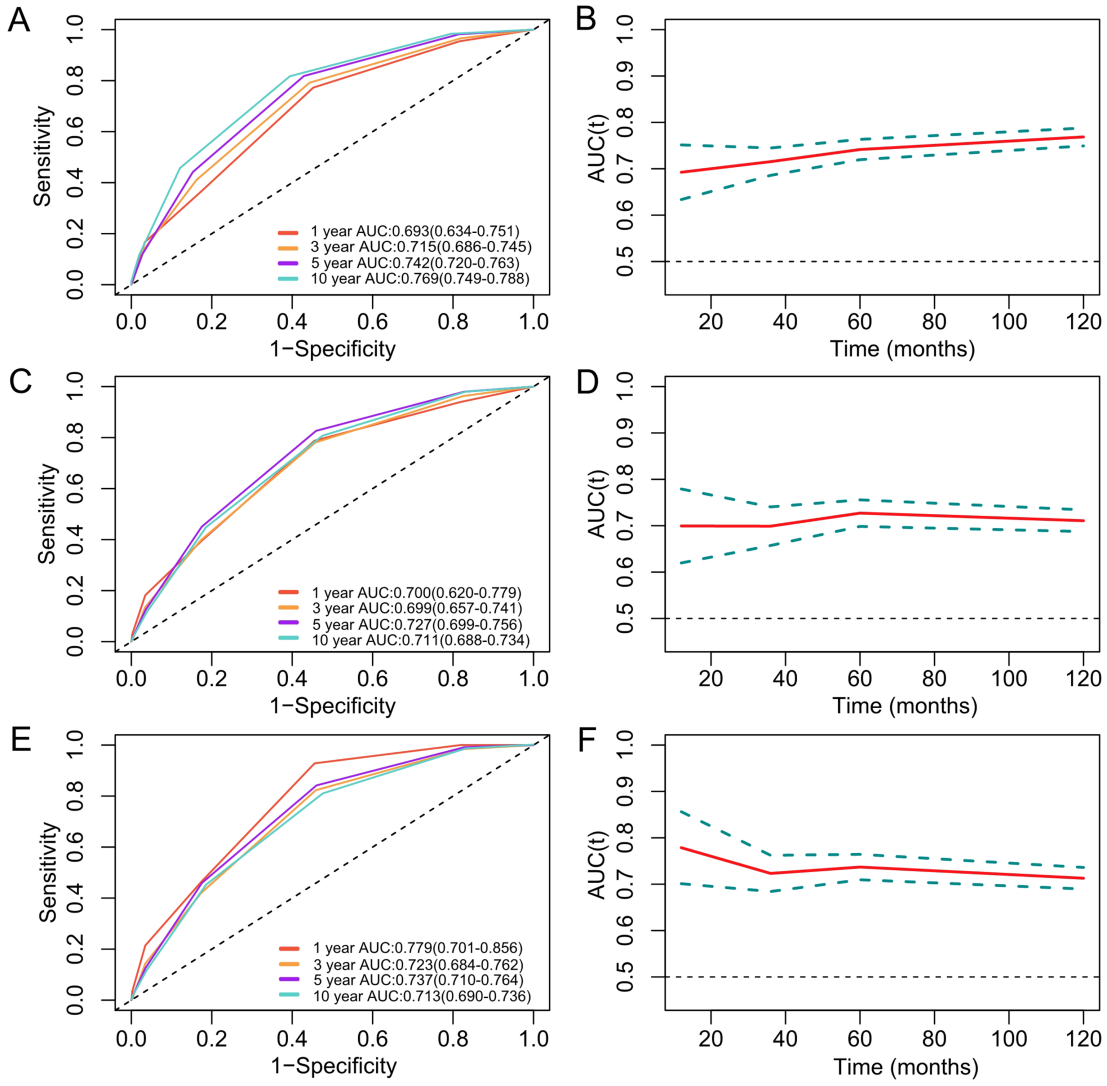
Time-dependent ROC curves and time-dependent AUC values (95% CI) for the prediction of all-cause **(A,B)**, cancer **(C,D)**, and CVD mortality **(E,F)** of MDS. ROC curves demonstrate MDS’s discrimination accuracy at 1-year (red), 3-year (orange), 5-year (purple), and 10-year (sky blue) follow-up. The time-dependent AUC **(B,D,F)** show MDS’s improving prognostic capacity over time, with the peak performance at 10-year follow-up for all-cause mortality (AUC = 0.769). AUC, area under the curve; CVD, cardiovascular disease; MDS, magnesium depletion score; ROC, receiver operating characteristic.

The prognostic significance of MDS for all-cause, cancer, and CVD death was assessed using time-dependent ROC analysis. For all-cause mortality, MDS demonstrated progressively improving prediction capacity with AUCs (95% CI) of 0.693 (0.634–0.751) at 1-year, 0.715 (0.686–0.745) at 3-year, 0.742 (0.720–0.763) at 5-year, and 0.769 (0.749–0.788) at 10-year follow-up (Figure 4). The AUC and 95% CI for cancer mortality of MDS at 1, 3, 5, and 10 years were 0.700 (0.620–0.779), 0.699 (0.657–0.741), 0.727 (0.699–0.756), and 0.711 (0.688–0.734). The AUC and 95% CI for CVD mortality of MDS at 1, 3, 5, and 10 years were 0.779 (0.701–0.856), 0.723 (0.684–0.762), 0.737 (0.710–0.764), and 0.713 (0.690–0.736). Collectively, MDS exhibited robust longitudinal predictive performance for all-cause, cancer, and CVD death outcomes.

### 3.4 Stratification and sensitivity analyses

To evaluate potential modifying effects of clinical characteristics on the observed associations, stratified analyses with interaction testing were performed (Supplementary Figures 2–4). The findings demonstrated consistent effects of MDS on mortality risk across most clinical features, including race, education level, BMI, and marital status (all interaction *p* values >0.05).

The observed correlation between MDS and mortality persisted in sensitivity analyses following the exclusion of participants deceased within 3 years post-baseline assessment (Supplementary Table 2). Furthermore, the statistical significance of this association remained robust when MDS was considered as a continuous variable (Supplementary Table 3).

## 4 Discussion

This study constitutes the comprehensive investigation examining associations between MDS and all-cause, cancer-specific, and CVD mortality among cancer survivors, drawing on both NHANES data and single-center records. Elevated MDS levels showed significant positive correlations with increased mortality risks across all endpoints. Time-dependent ROC curve analysis confirmed MDS as a robust predictor of all-cause, cancer-related, and CVD mortality. Subgroup and sensitivity analyses consistently reinforced these associations. To our knowledge, this work provides the first evidence establishing MDS as a mortality risk factor in cancer survivor populations.

Previous studies have explored the association between MDS and various health outcomes. Higher MDS is positively correlated with hypertension (20), diabetes (21), and metabolic syndrome (22) prevalence. MDS has also been found to be associated with lower serum levels of the anti-aging protein Klotho in middle-aged and older adults, suggesting a potential link to accelerated aging (23). MDS has been shown to be a better predictor of magnesium deficiency than serum magnesium alone and is associated with increased systemic inflammation and CVD mortality, particularly in individuals with low magnesium intake (9). These findings highlight the importance of MDS as a valuable indicator of magnesium status and its potential implications for various health conditions. No prior studies have examined the relationship between MDS and mortality in cancer patients. This study investigates the associations of MDS with all-cause, cancer-specific, and CVD mortality to clarify their complex interplay.

The relationship between magnesium and cancer is complex and not fully understood. While magnesium deficiency is associated with increased risk of certain cancers in epidemiological studies, experimental models show both anti- and pro-tumor effects of low magnesium (24). Magnesium plays crucial roles in cellular processes, including DNA replication and repair (25). In animal models, magnesium deficiency inhibits primary tumor growth but enhances metastasis (26). Magnesium supplementation generally inhibits carcinogenesis, possibly by reducing carcinogen binding to cells and DNA (27). However, conflicting results from various studies highlight the need for more research to elucidate the precise mechanisms linking magnesium and cancer.

The eGFR serves as a biomarker predictor for increased CVD and cancer risk, with lower eGFR values associated with progressively higher risk of both conditions. Go et al. (28) studied 1,120,295 adults and found graded increases in cardiovascular risk as eGFR declined below 60 mL/min/1.73m^2^, with HRs ranging from 1.4 (eGFR 45–59) to 3.4 (eGFR <15). For cancer, Xu et al. analyzed 719,033 Swedes and identified a U-shaped relationship, with eGFR 30–59 showing a hazard ratio of 1.08 for cancer incidence (29). The interaction is particularly concerning in cancer patients. It was found that among 25,274 adults, those developing cancer with even mild renal impairment (eGFR 70–99) had 2.7–2.9 times greater risk of cardiovascular events and mortality compared to cancer-free individuals (30). Proton pump inhibitors are linked to CVD through magnesium-mediated mechanisms. Experimental evidence in rats demonstrated that PPIs increase ventricular arrhythmias through magnesium depletion, which increases superoxide production and sympathetic hyperinnervation (31).

Existing researches present conflicting evidences regarding the association between serum magnesium levels and cancer patient outcomes. Magnesium’s role in cell growth regulation and immune function has been highlighted, with evidence suggesting that hypomagnesemia may increase cancer risk and worsen prognosis in solid tumors (32). Higher magnesium levels were associated with improved progression-free survival and overall survival in patients receiving immune checkpoint inhibitors for various cancers (33). However, a study on metastatic urothelial carcinoma patients found no significant association between magnesium levels and outcomes or adverse events with atezolizumab treatment (34). The divergent findings across these studies likely stem from variations in tumor types and therapeutic regimens, or from relying solely on serum magnesium measurements rather than more precise assessment methods like the MDS. It underscores that further researches are needed to fully elucidate its role across different cancer types and treatments.

Beyond its specific role in quantifying magnesium-depleting conditions, the MDS likely reflects broader systemic vulnerability, particularly among older cancer survivors. Our data show that participants with an MDS ≥ 3 were significantly older, with average ages of 72.45 versus 49.17 years in the NHANES cohort and 76.3 versus 53.1 years in the single center cohort. They also exhibited a higher burden of comorbidities. In older adults, higher MDS correlates with increased frailty prevalence and elevated all-cause and CVD mortality risk, with HRs reaching 2.751 for all-cause mortality in the highest MDS category (35). Among CKD patients, MDS > 2 independently predicts long-term mortality, with associations strongest in those with inadequate magnesium intake (36). These findings suggest MDS captures not only magnesium-depleting conditions but also reflects broader systemic health vulnerability across various populations and disease states.

Our study offers several key strengths that enhance its scientific and clinical practicability. The study introduces the MDS, a clinically practical tool that integrates multiple accessible factors to assess magnesium status, offering a significant advance over traditional serum magnesium measurements. Leveraging nationally representative NHANES data with a robust sample size (*n* = 3,528) and hospitalized persons (*n* = 473), the findings are generalizable and statistically rigorous. Additionally, the comprehensive analytical approach further underscores the rigor of the methodology, including weighted multivariate cox regression, competitive risk model constructed using Fine-Gray’s test, time-dependent ROC, and sensitivity analysis. These methods collectively validate the observed associations while maximizing the minimization of confounding factors. However, this study has several limitations. First, the current study assessed MDS solely at baseline without evaluating its dynamic changes. During follow-up, a patient’s MDS can change as the four component items vary. Neither cohort, however, captured post-baseline MDS, so we cannot rule out the possibility that such changes influenced outcomes. At present, no consensus exists on the timing or frequency of dynamic MDS assessment; given these objective constraints, obtaining longitudinal data was beyond the scope of the current study. We look forward to future investigations that will refine the definition and monitoring of MDS. Second, we did not conduct separate analyses stratified by cancer type among survivors due to the limitation of sample size, which may introduce bias due to potential variations in survival durations across different malignancies. Furthermore, the cross-sectional nature of present study also precludes establishing causality.

## 5 Conclusion

This study robustly demonstrates that the MDS serves as a significant independent prognostic indicator for mortality risk among cancer survivors. Using nationally representative NHANES data and validating findings in a separate hospital cohort, we found that higher MDS scores were strongly and monotonically associated with increased risks of all-cause, cancer-specific, and cardiovascular disease mortality, even after comprehensive multivariable adjustment. Therefore, integrating MDS assessment into routine survivorship care offers a valuable, clinically feasible tool for identifying high-risk cancer survivors.

## Data Availability

The original contributions presented in the study are included in the article/Supplementary material, further inquiries can be directed to the corresponding author.
