# Supplementary material for: Magnesium depletion score is a risk factor for all-cause, cancer and cardiovascular disease mortality in cancer survivors: evidence from two prospective cohort studies
Source: Front Nutr. 2025 Oct 31;12:1674062. doi: 10.3389/fnut.2025.1674062 (PMC12616742; doi:10.3389/fnut.2025.1674062)
Supplement: Supplementary file 1 [file Table_1.DOCX]

**Supplementary Material:**

Supplemental Table 1. Definitions and classification of covariates.

Supplemental Figure 1. The weighted multivariable cox regression models analysis on the association between different MDS groups and all-cause, cancer, and CVD mortality.

Supplemental Figure 2. Subgroup analysis on the association between MDS and all-cause mortality.

Supplemental Figure 3. Subgroup analysis on the association between MDS and cancer mortality.

Supplemental Figure 4. Subgroup analysis on the association between MDS and CVD mortality.

Supplemental Table 2. Weighted Multivariate Cox analyses (excluding participants who died within 3 years of follow-up) of the association between different MDS groups and the risk of all-cause, cancer, and CVD mortality.

Supplemental Table 3. Weighted Multivariate Cox analyses (excluding participants who died within 3 years of follow-up) of the association between MDS (continuous) and the risk of all-cause, CVD, and cancer mortality.

**Supplemental Table 1. Definitions and classification of covariates.**

| **Variables** | **Definition and classification** |
| --- | --- |
| Smoking status | Never: smoked less than 100 cigarettes in life  Former: smoked more than 100 cigarettes in life and smoke not at all now  Now: smoked moth than 100 cigarettes in life and smoke some days or every day |
| eGFR | Method: CKD_EPI_Scr_2021 |
| Diabetes mellitus | Doctor told you have diabetes;  Glycohemoglobin HbA1c(%) ≥ 6.5,  Fasting glucose (mmol/l) ≥ 7.0,  Random blood glucose (mmol/l) ≥ 11.1,  Two-hour OGTT blood glucose (mmol/l) ≥ 11.1,  Use of diabetes medication or insulin. |
| Hypertension | The diagnostic criteria consist of self-reported hypertension history, the utilization of antihypertensive medication, a systolic blood pressure (SBP) ≥ 140mmHg, or a diastolic blood pressure (DBP) ≥ 90mmHg |

**Supplemental Figure 1. The weighted multivariable cox regression models analysis on the association between different MDS groups and all-cause, cancer, and CVD mortality.
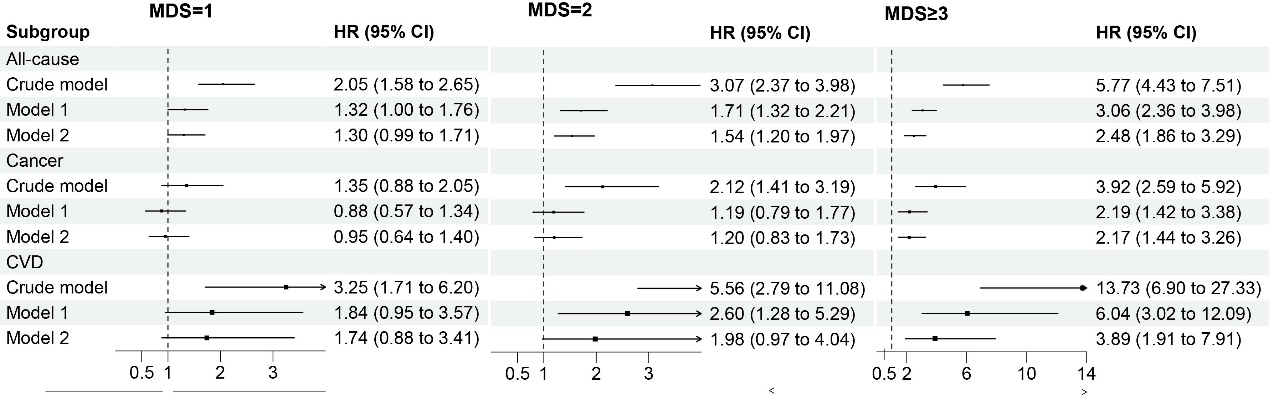
**

Abbreviations: CVD, cardiovascular disease; MDS, magnesium depletion score; HR, hazard ratio.

**Supplemental Figure 2. Subgroup analysis on the association between MDS and all-cause mortality.**


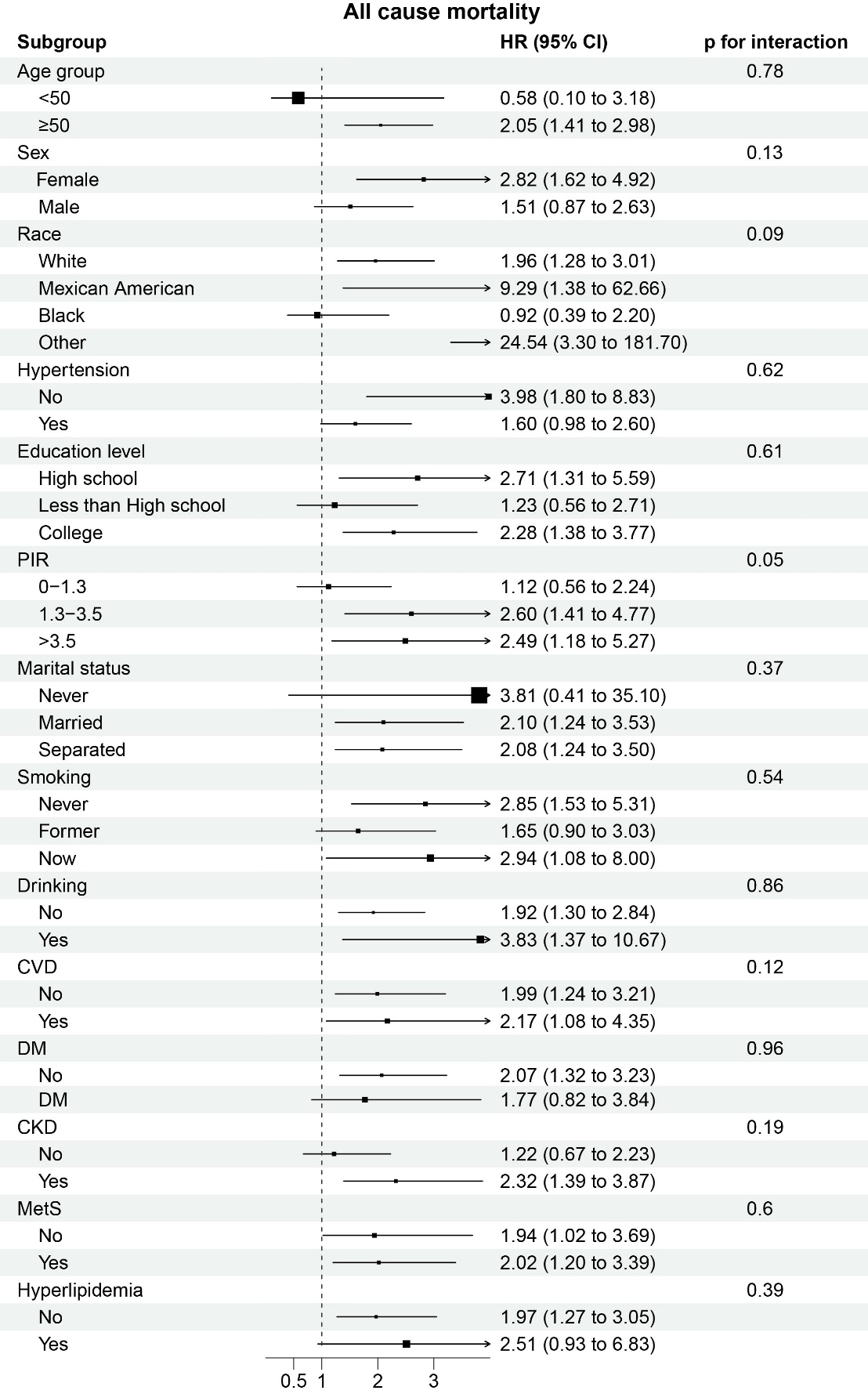


Abbreviations: BMI, body mass index; CKD, chronic kidney disease; CVD, cardiovascular disease; DM, diabetes mellitus; MetS, metabolic syndrome; MDS, magnesium depletion score; HR, hazard ratio; PIR, poverty-income ratio.

**Supplemental Figure 3. Subgroup analysis on the association between MDS and cancer mortality.**


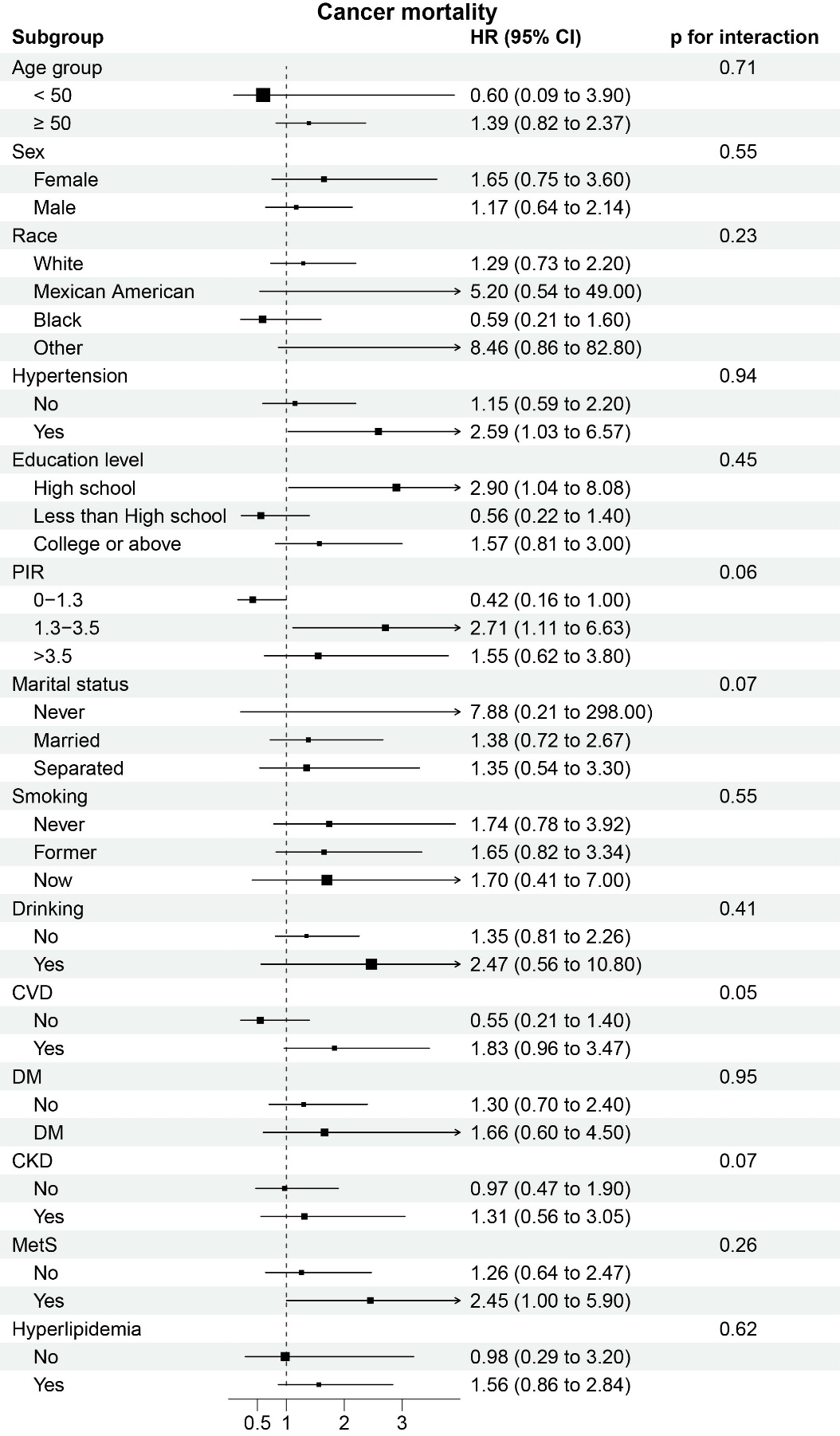


Abbreviations: BMI, body mass index; CKD, chronic kidney disease; CVD, cardiovascular disease; DM, diabetes mellitus; MetS, metabolic syndrome; MDS, magnesium depletion score; HR, hazard ratio; PIR, poverty-income ratio.

**Supplemental Figure 4. Subgroup analysis on the association between MDS and CVD mortality.**

**
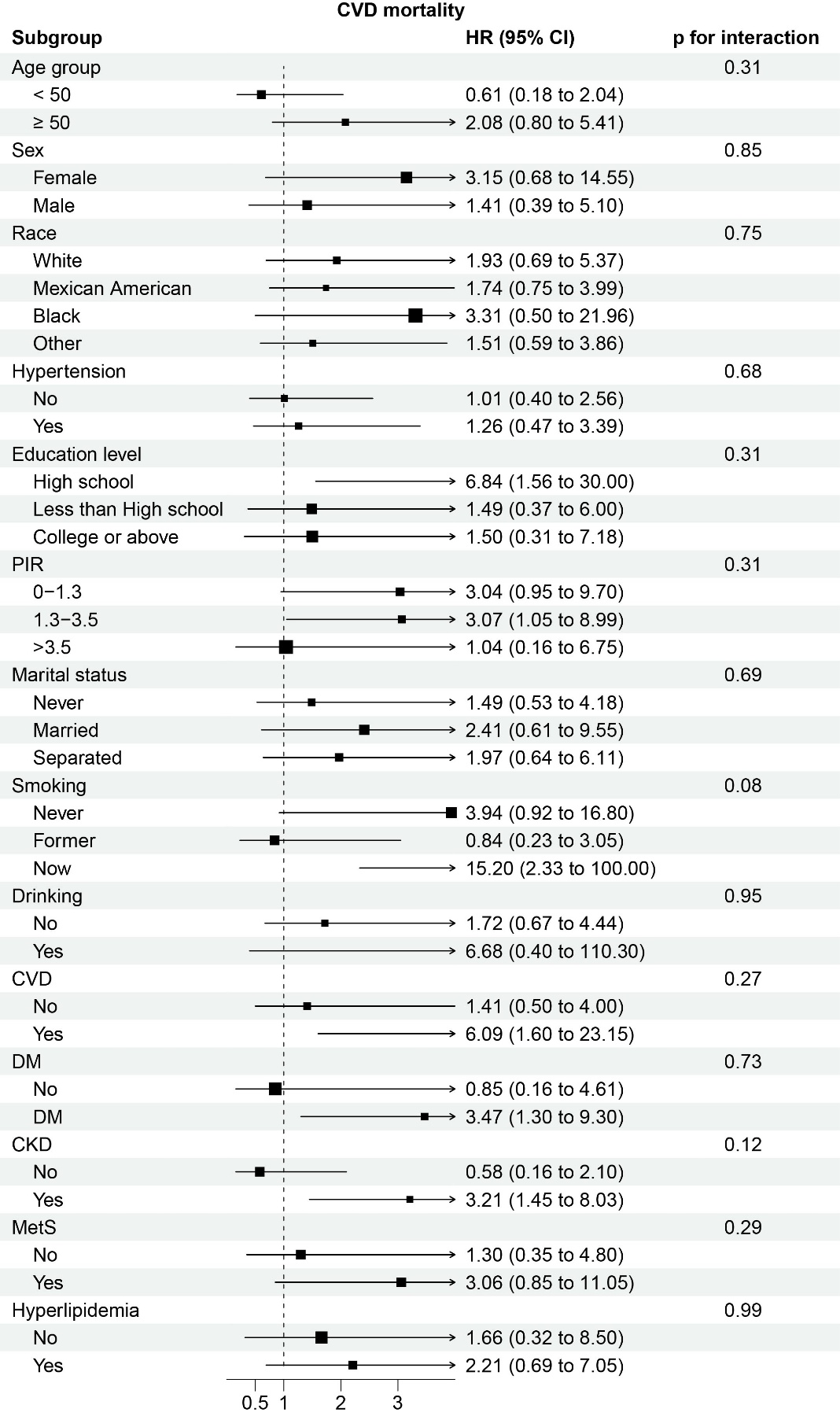
**

Abbreviations: BMI, body mass index; CKD, chronic kidney disease; CVD, cardiovascular disease; DM, diabetes mellitus; MetS, metabolic syndrome; MDS, magnesium depletion score; HR, hazard ratio; PIR, poverty-income ratio.

**Supplemental Table 2. Weighted Multivariate Cox analyses (excluding participants who died within 3 years of follow-up) of the association between different MgDS groups and the risk of all-cause, cancer, and CVD mortality.**

| **Cause of death** | MDS groups, HR (95% CI) | | | | | p | p for trend |
| --- | --- | --- | --- | --- | --- | --- | --- |
|  | 0 | 1 | 2 | | ≥3 |  |  |
| All-cause | | | |  |  |  |  |
| Crude model | Ref | 2.61(1.85, 3.70) | 3.81(2.70, 5.37) | | 7.28(5.14,10.32) | <0.0001 | <0.0001 |
| Model 1 | Ref | 1.66(1.14, 2.42) | 2.04(1.42, 2.92) | | 3.69(2.59, 5.26) | <0.0001 | <0.0001 |
| Model 2 | Ref | 1.63(1.12, 2.37) | 1.87(1.33, 2.64) | | 3.00(2.05, 4.40) | <0.0001 | <0.0001 |
| Cancer | | | |  |  |  |  |
| Crude model | Ref | 1.62(0.86,3.07) | 2.68(1.50,4.79) | | 4.54(2.50,8.23) | <0.0001 | <0.0001 |
| Model 1 | Ref | 1.03(0.55, 1.95) | 1.41(0.81, 2.48) | | 2.37(1.29, 4.34) | 0.01 | <0.001 |
| Model 2 | Ref | 1.17(0.67, 2.05) | 1.61(0.97, 2.66) | | 2.65(1.54, 4.57) | <0.001 | <0.0001 |
| CVD | | | |  |  |  |  |
| Crude model | Ref | 3.14(1.56, 6.33) | 5.20(2.47,10.97) | | 12.02(5.62,25.73) | <0.0001 | <0.0001 |
| Model 1 | Ref | 1.77(0.86, 3.64) | 2.39(1.11, 5.14) | | 5.21(2.42,11.21) | <0.0001 | <0.0001 |
| Model 2 | Ref | 1.69(0.81, 3.55) | 1.86(0.85, 4.08) | | 3.38(1.54, 7.42) | 0.002 | <0.001 |

Crude model: unadjusted;

Model 1: adjusted for age, sex, race, education level;

Model 2: further adjusted for marital status, PIR, BMI, smoking, drinking, hypertension, hyperlipidemia, DM, CVD, CKD, and MetS.

Abbreviations: BMI, body mass index; CI, confidence interval; CKD, chronic kidney disease; CVD, cardiovascular disease; DM, diabetes mellitus; MetS, metabolic syndrome; MDS, magnesium depletion score; HR, hazard ratio; PIR, poverty-income ratio; Ref, reference.

**Supplemental Table 3. Weighted Multivariate Cox analyses (excluding participants who died within 3 years of follow-up) of the association between MDS (continuous) and the risk of all-cause, cancer, and CVD mortality.**

| **Cause of death** | MDS (Continuous),  HR (95% CI) | p |
| --- | --- | --- |
| All-cause | |  |
| Crude model | 1.66(1.55,1.78)  1.43(1.33,1.54)  1.34(1.23,1.45) | <0.0001 |
| Model 1 |  | <0.0001 |
| Model 2 |  | <0.0001 |
| Cancer | |  |
| Crude model | 1.57(1.39,1.78)  1.35(1.16,1.57)  1.37(1.19,1.58) | <0.0001 |
| Model 1 |  | <0.0001 |
| Model 2 |  | <0.0001 |
| CVD | |  |
| Crude model | 1.89(1.61,2.22)  1.60(1.35,1.90)  1.37(1.15,1.62) | <0.0001 |
| Model 1 |  | <0.0001 |
| Model 2 |  | <0.001 |

Crude model: unadjusted;

Model 1: adjusted for age, race, education level, marital status, and PIR

Model 2: further adjusted for BMI, smoking, drinking, marital status, hypertension, hyperlipidemia, DM, CVD, CKD, and MetS.

Abbreviations: BMI, body mass index; CI, confidence interval; CKD, chronic kidney disease; CVD, cardiovascular disease; DM, diabetes mellitus; MetS, metabolic syndrome; MDS, magnesium depletion score; HR, hazard ratio; PIR, poverty-income ratio.
